# Building a robotic self

**DOI:** 10.1098/rstb.2025.0166

**Published:** 2026-11-10

**Authors:** Martin V. Butz, Verena V. Hafner, Pablo Lanillos

**Affiliations:** ^1^ Department of Computer Science and Department of Psychology, Eberhard Karls Universität Tübingen, Tübingen, Germany; ^2^ Department of Computer Science, Humboldt-Universität zu Berlin, Berlin, Germany; ^3^ Cajal Neuroscience Center, Spanish National Research Council (CSIC), Madrid, Spain; ^4^ Donders Institute for Brain, Cognition and Behaviour, Nijmegen, The Netherlands

**Keywords:** synthetic self, agency, embodied AI, predictive coding

## Abstract

I wake up in the morning, I see my hand and I know it is me. This seemingly simple skill lies at the foundation of our agentive selves. We assume that the development of the self, in both biological and synthetic agents, is grounded in sensorimotor embodiment and inductive learning biases, as well as in the principle of predictive coding. Here, we explore the core inductive biases and process components for building a robotic minimal self. We propose that the formation of a hierarchical, generative model of the self is driven by active inference processes that interactively infer both an endogenous contextual frame and behaviour within this frame. Accordingly, experimental implementations in humanoid robots show how body ownership and agency can develop as foundational computational components. This work on building a robotic minimal self outlines future research directions in mechanistic modelling of the development of an artificial self. To probe the depth of this development, we sketch out embodied Turing tests that challenge complex problem-solving skills, as well as social intelligence and theory of mind. We suggest that uncertainty resolution and the social domain may be promising avenues for further research in the near future.

This article is part of the discussion meeting issue ‘From being to identity: analysis and synthesis of the self’.

## 1. Introduction

*The self is a repeatedly reconstructed biological state.* Antonio Damasio [1, p. 227]

Investigating the mechanisms of self-construction is fundamental to understanding human cognition and intelligence. We address this challenge using the synthetic approach [2–4], which tries to build artificial agents that replicate human psychological phenomena [5], function [6] and even brain mechanisms [7]. It allows us to investigate how the self may develop from a mechanistic and embodied perspective by using robots. Theoretical principles (functions) are translated into computational models (mathematical framework) that are deployed into physical hardware (robot). The robots’ behaviour is then evaluated by an external observer, for example, via the embodied Turing test.

We adhere to the Bayesian brain hypothesis, which postulates that brains develop bounded, body-grounded world models from sensorimotor experiences [8]. These world models enable the invocation of predictive and anticipatory mechanisms. As a result, world models constitute the foundation of self-construction. Endorsing the Bayesian account provides the tools for a mathematical implementation via, for example, the active inference account to perception and action [9,10]. However, in order to learn world models from sensorimotor experiences fast and efficiently, important decisions need to be made about what prior knowledge should be incorporated into the artificial intelligent system. The inclusion of inductive biases—like an artificial evolutionary push—can shorten the process of learning suitable world models. The assessment of the resulting synthetic self is as important as the computational model. Such assessments have to fulfil a set of prerequisites [11]. They should also be incrementally validated through comparison with human data (e.g. body and perceptual illusions [12] and self-tests [13]), and by observing behaviour and emergent self-related signatures. All this does not seek to create a human-equivalent self in machines. Rather, the goal is to explore and understand the long journey towards self-construction and cognition itself.

Here, we describe the possibility of constructing a robotic minimal self through three blocks: what computational mechanisms should be embedded and how can they be described mathematically (§2), how can we deploy these models into robotic systems (§3) and how can we assess and validate the presence of a minimal self and what are current key challenges (§4).

## 2. Developing a synthetic self

*But how the I that I think is to differ from the I that intuits itself […] is no more and no less difficult than how I can be an object for myself in general and indeed one of intuition and inner perceptions.* Immanuel Kant (translation from [14])

We propose that the development of a self—both in biological agents, including us humans, and in synthetic software and robotic agents—is grounded in two foundational components: sensorimotor embodiment and inductive learning biases [2,3,15]. Together, these two components determine how and to which extent agents learn, plan and reason.

In this section, we first discuss the importance of inductive biases and emphasize that such biases are grounded in the embodiment of an agent. We then highlight that embodied inductive biases offer a basis for interpretation. That is, to effectively, flexibly and adaptively interact with our world, we argue that the stream of sensorimotor experiences needs to be abstracted into concepts and events that are embedded into self-constructed contextual frames. Next, we provide a mathematical framework of the proposed principles that offers a rigorous formalism of the discussed concepts.

### (a) Embodiment and inductive biases

Embodiment emphasizes that the sensory and motor systems, which are part of the body of the agent with its body-particular schematic structure (joints, limbs, their relative arrangements, their strengths, etc.), provide and generate the sensorimotor data. Sensorimotor data are the only information that is available to the agent (i.e. its brain) to develop internal knowledge and state estimates. It should be emphasized, though, that these data only give a glimpse at the true state of the environment. Despite us having the phenomenological impression of perceiving the complete world around us—in colour—our sensors do not provide such detailed information. Even when considering the rather immediate surroundings of the agent, the glimpse is rather restricted. For example, we typically do not know how a table looks from the bottom. Similarly, we do not perceive all sides of an object or the complete body of another person, but seamlessly assume that it is complete.

Additionally, it is well-known that we cannot process all the (glimpsed at) information that is in principle available. Change blindness experiments [16,17] illustrate that we strongly abstract away from the sensory information. In the experiments, participants look at a photo and are asked to identify a change. When the change is masked by a well-timed distractor, such as a flicker, we typically do not become aware of the change, even if it is rather noteworthy. However, once we have detected the change (once we have somewhat accidentally directed our attention towards the thing that changes), it springs into our eyes and we cannot believe that we had missed it before. Similarly, ever since the foundational work on eye fixations and saccades by Yarbus [18] in the 1960s, it is well-known that we process the scene that we view in a very task-oriented manner. Inattentional blindness experiments illustrate this fact most impressively: we overlook a gorilla walking through the scene when we are asked to count the passes of a basketball team. We even overlook the identity change of a person when confronted with a stranger and when the switch of the person happens out of view, like under a counter or behind a large board that distracts attention [19,20].

As a part of embodiment, morphological intelligence [3,21] develops the basic wiring to process this information and provides core reflexes to bootstrap ideomotor development.^1^ Building on these foundations, progressively generalized and abstracted knowledge structures develop—shaping our world models. These world models appear to come in the form of generative and predictive structures [8,9,23–25]. They can be partitioned into (i) encoding–decoding pipelines that form latent conceptual spaces, which offer a variety of conceptual state space encodings; (ii) temporal predictive models that encode the intuitive knowledge how world and bodily dynamics unfold over time; and (iii) procedural knowledge in the form of behavioural policies, which generate priors over action spaces.

To bootstrap this knowledge development further, besides morphological intelligence, evolution has developed computational inductive information processing and learning biases. These biases streamline and focus cognitive development. In machine learning, such biases are well-known to be key to scalability, data-efficiency and targeted structural learning in artificial agents [26]. These biases can be implemented within the computational units that constitute the developing world model of the agent.

It should be acknowledged that successful development depends on the interactions between both of these components. Embodiment and morphological intelligence shape the input data, including its dynamics, and—in interaction with the body and the outside environment—determine the generation of the data. Inductive biases then shape the cognitive development of progressively more abstract internal encodings. We now discuss which core inductive biases may be identified that shape this cognitive development—including the development of one’s own self.

### (b) Predictive concepts, events and contextual frames

The insights from the embodiment side imply that our minds do not think within the full external environment. That is, we do not represent the complete external environment, and we do not plan within a representation of the complete external environment. Equally, we do not think on the sensory level. Rather, we selectively encode those aspects of the world that seem behaviourally relevant to us. We embed these aspects into internal distributed state estimates. The nature of these state estimates shapes how we think about the world and about ourselves within the world. A three-partite hierarchy has been proposed as a foundational structural schema characterizing internal distributed state estimates: concepts, events and contexts [27].

A state estimate is principally encoded via a network of selectively activated conceptual state spaces [28,29]. Conceptual spaces encode modal and amodal features, such as object properties, relations between such properties and between entities, as well as spatio-temporal relations. Concepts are extracted from the sensorimotor information and appear generative in nature. Similar to an autoencoder pipeline, conceptual spaces encode information in a generative predictive manner, that is, top-down predictions anticipate sensory data and the comparison between those predictions and the actual sensory data is propagated back upwards [25].

A state estimate furthermore binds these conceptual encodings into entity encodings, including objects [30,31], other agents and the agent’s own body [13]. Furthermore, conceptual as well as entity encodings are put into relation to each other in space and time, yielding event-predictive encodings [32–35]. Schemata of such encodings yield processing templates and allow the generalization of particular concepts to related situations. Events as well as episodic sequences thereof thus segment objects and their interaction dynamics episodically, enabling the invocation of self-motivated epistemic and goal-directed behaviour [36,37] as well as the selective replay and generalizations away from the episodic structure into cognitive maps and world models [27,38].

While predictive encodings of concepts and events constitute our experienced reality, possibly the most important component in our cognitive apparatus has been largely overlooked for decades. Our minds appear to infer endogenous *contextual frames* within which we process concepts and events. We appear to infer these frames from our sensorimotor experiences on the fly and fully seamlessly—solving the frame problem [2,39]. As we have discussed above, embodiment and morphological intelligence provide only a selective glimpse at the outside environment. This glimpse itself can be understood as a sensorimotor contextual frame. Combined with selective conceptual abstractions and event-predictive encodings, the grounded contextual frame becomes abstract as well. As argued elsewhere [27], we propose that it is the inference of endogenous contextual frames that determines the aspects of the environment that are currently under our cognitive disposition. That is, we direct our attention, think, plan and infer behaviour within the currently activated contextual frame. Yet in other words, the contextual frame activates those conceptual spaces, types of entities, spatial outlines and event-predictive schemata that we use to perceive and act.

Overall, we propose that the selective activation of contextual frames constitutes our current endogenous reflection of the outside environment, including possible future continuations of it. The contextual frame selectively activates state space representations and state transition encodings. Combined with the actual sensorimotor data, the combination of concepts and events together with the contextual frame, within and relative to which the concepts and events are processed, constitutes our experiences—offering the data for the formation of episodic and long term memory [15,27,40]. To control our behaviour and attention, the contextual frame offers the basis. That is, it offers the state space and world model structure for reasoning, planning and decision-making. For example, a counterfactual argument can only be made within a boundedly complex image of the actual reality, which we term the contextual frame. We propose that it is this contextual frame that offers the basis for the formation of an active self and will be key for the implementation of a synthetic self.

### (c) Mathematical formalization

Building on the contextual frame theory, we formalize the agentive self as a contextualized partially observable Markov decision process (C-POMDP). Our proposal is related to a recent explicit formalization of the body of an agent separated from the environment by means of an enriched POMDP and the mentioning of Meta ePOMDPs, which switch between ePOMDPs [41]. In our case, we do not explicitly distinguish between agent and environment but propose that this distinction emerges. Rather, our formalism emphasizes that an agent actively infers both the current contextual frame c and its actual behaviour (including covert attention and overt motor actions a). The agent has only partial access to the actual state of the environment via observations o, where the environment includes the state of the world and the body x={w,b}. Furthermore, the agent can perform actions a that influence the environmental dynamics over time.^2^ This system’s world model knowledge m(⋅) can be fully described by a graphical model that encodes the likelihood of an observation o given the current endogenous state estimate z: p(o|z,c), the transition model $p(z_{t+1}|z_{t},a_{t},c)$ and a behavioural policy π(a|z,c). As the agent has access to the environment only via its embodied sensorimotor interface, it generates endogenous, latent state estimates z as predictive codes that explain away observations o. The transition model constitutes the state transitions that are currently deemed relevant, as specified by the contextual frame. Similarly, the action policy is focused onto those actions that are relevant given the current contextual frame.

Planning, reasoning and decision-making then unfold within the C-POMDP. As the context essentially opens up the aspects of reality that are currently under consideration, it naturally opens up the frame (i) to take different perspectives onto the observed and generated events as well as (ii) to reason in a counterfactual manner about eventive interactions. To accomplish this, a contextual frame needs to be sufficiently stable over an extended period of time. Meanwhile, though, the contextual frame needs to be malleable as it otherwise may sometimes be overly restricted. This can be vividly noticed when we have a Eureka or Aha moment [42], in which case the context was overly restricted at first and then widens up enabling the finding of a solution to a task. The hardest problem that remains widely open, however, is how to infer the contextual frame itself at any point in time. In the remainder of this article, we leave this last hard problem for future work.

### (d) Summary and implications

To summarize, we propose that predictive coding and embodied sensorimotor data form the basis of our cognitive selves and thus are well-suited candidates for the construction of a synthetic self. Because self-induced effects on the agent’s own body are easiest to predict, reafferent sensorimotor models will be learned first [43]. Once the effects on the own body are understood, effects on the body-surrounding environment come under consideration. As a result, the learning of conceptual spaces as well as schemas of entities, events and contextual frames commences. The generation of episodic experiences is thus realized by means of concept–event–contextual frame encodings, where concepts, entities and events are embedded in self-inferred contextual frames. An inferred contextual frame essentially enables us to focus our experiences on those aspects of the outside environment and our body that we currently deem relevant. As a result, well-predictable effects of actions will be pre-empted by our contextualized prediction dynamics and will thus be seamlessly integrated into our experiences. On the other hand, unexpected and less predictable observations will be harder to process, trigger surprise and will be less seamlessly integrated into our episodic experiences [35,44].

## 3. A robotic minimal self

*We often consciously will our own actions. This experience is so profound that it tempts us to believe that our actions are caused by consciousness.* D. M. Wegner [45, p. 65]

To endow robots with a self, we first need to distinguish between the awareness process that we associate with biological entities and the necessary components that settle the pillars of a functional minimal self [46]. Perception and action are deeply intertwined processes that also operate in an unawareness regime [45], producing unconscious inferences in perception and action [47–49]. Thus, we may start excluding phenomenal experience when discussing the robot self, and focus instead on the basic principles of sensorimotor integration—where embodiment and morphological intelligence enable conceptual grounding—for the formation of a minimal self. For a comprehensive review on the use of robots to investigate the self beyond the minimal self, please refer to Prescott *et al.* [50]. This functional or sensorimotor minimal self has been studied from an interdisciplinary perspective in the Priority Programme ‘The Active Self’.^3^ The programme contributed both to understanding the underlying mechanisms of the human minimal self [51] and to investigating the prerequisites for a synthetic self [11,52]. It has been pointed out that a minimal robotic self should embed those mechanisms that functionally cover at least the following two questions [53]: (i) *is this my body?* (body ownership) and (ii) *did I do it?* (agency).

One of the basic principles to model a minimal self is the capacity to distinguish self-generated signals from externally generated ones [54]. This approach reduces the problem to: *am I generating those sensations?* Most mechanisms proposed to resolve this question are based on the ‘comparator’ model [55] that explains how actions are monitored, which is related to the reafference and ideomotor principles [24,43,56,57] that explain how actions are chosen. The model postulates that the brain generates an internal prediction of the effects of an initiated movement, that is, of reafferences. If those predicted reafferences match with the actually perceived movement, authorship is strengthened. Measurements and factors of self-action attribution are temporal binding [58], predictability of the signal [59], and causality of the effect produced [60,61]. Congruency is also an important factor for body ownership. For instance, in the rubber hand illusion (RHI) experiment [62], the spatial congruency and the synchronicity of the visuo-tactile stimulation, the spatial contingency between felt and seen strokes and the plausibility of the rubber hand and rubber arm’s orientation are essential to generate the feeling of embodying an external limb [63–65]. An active/moving version of the RHI also includes self-action attribution [66].

In a robotic setting, this means that the robot needs to (i) learn sensorimotor contingencies, i.e. mappings between causally related sensory and motor information [67], (ii) predict motor-induced changes in the sensory information [13], and (iii) establish causality between body and external cues [68].

### (a) Is this my body?

Knowing that our body belongs to us can be further split into: (i) how we localize our body in space using our body schema, i.e. the internal representation of the body, including the position of its limbs and their relation to the surrounding space, and (ii) how we recognize our body through the body image (e.g. visual appearance) [69]. We motivate the following synthetic self implementation on a robot under two well-known experimental paradigms: the RHI and the mirror self-recognition (MSR) test [70]. In the MSR (also known as the red mark test), a coloured mark is secretly applied to the subject’s face, and the subject is placed in front of a mirror. The test is passed if the subject can locate the mark on their own body. The MSR draws a connection between the body image and the body schema and explores the relevance of sensorimotor contingencies. The RHI implies that the human sense of body ownership is adaptive itself, as it can be temporarily altered by multi-sensory cues that imply that the rubber hand must be the own hand.

#### (i) Body localization and the rubber hand illusion effects

The robot brain needs to keep track of a body posture or schema [71] by fusing information from all available sensory inputs. Following the Bayesian brain assumption [9,72], and using the C-POMDP mathematical formalization described above, we define body localization as inferring the body state x given the sensory input o: p(x|o,c), where the context encodes the presumed current body schema and image. Unfortunately, finding the true exact distribution of x is not possible in almost all real applications. Thus, instead of computing the exact posterior, we transform the problem into approximating an auxiliary internal belief q(z) to the real posterior distribution given the observations: q(z)→p(x|o,c). This endogenous belief is a tractable and known density function, such that we can perform inference. To compute it, we transform Bayesian inference into an optimization problem where the solution is the internal representation that best fits the observation data: $z=\arg\min_{z}D_{\mathrm{KL}}[q(z|c)||p(x|o,c)]$. The Kullback–Leibler divergence ($D_{\mathrm{KL}}$) measures the distance between the internal belief and the real state posterior of the system. This distance is zero when both distributions are the same. However, this problem is usually as complex as computing the exact posterior. Hence, a bound on this metric called variational free energy (F) or (negative) evidence lower bound is used instead.^4^ The goal is to minimize the distance of the internal belief and the joint distribution (q(z|c)→p(x,o|c)), which is fully defined by the observation likelihood and the transition function of the C-POMDP. Thus, we arrive at the final body state estimator:

(3.1) $$\begin{array}{r} \text{Perception}\;z=\arg\min_{z}F(o,z|c). \end{array}$$

One common use of F when states, actions and time are continuous ([73]—see [74] for a practical derivation and deployment in a humanoid robot) is the famous differential equation proposed by Friston [9] and Friston *et al.* [75] which formalizes body state estimation updates under the predictive processing account: $\dot{z}={\partial_{z}\epsilon }^{T}\Pi \epsilon$. In practice, the belief of the body state is corrected given current precision (inverse variance) weighting (Π) and prediction error (ϵ), e.g. difference between the sensory input and the predicted input given the internal state [73]. Respective precision estimates describe how much multi-sensory information (proprioception, vision, touch, etc.) contributes to body localization. It is modulated by the noise associated with each modality. For instance, in humans, visual input is typically relatively precise while proprioception is noisy. In robots, this situation is typically inverted.

Figure 1A describes the computation of the free energy F (equation 3.1) depending on the position of the hand given proprioceptive and visual feedback. This mechanism should also account for the reported mislocalizations in body illusions. In particular, we validated the proprioceptive drift that appears in the RHI. When subjected to multi-sensory conflicts in the form of visuo-tactile stimulation, the localization of the hand should be biased towards the fake hand. Figure 1B describes the obtained proprioceptive drift when replicating the RHI in a robot, comparing it to data from human participants.

**Figure 1.**
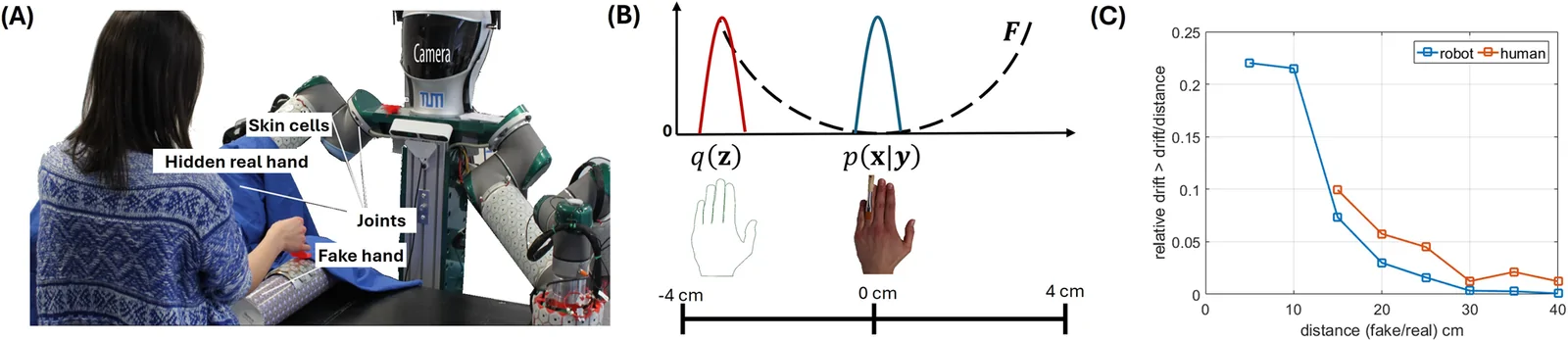
Flexible body estimation. (A) Replicating the RHI in a robot with visual, proprioceptive and tactile information. (B) Free energy value depending on the visual position of the fake hand. The approximation of the hand location belief to reality given manipulated sensory information produces the perceptual drift (mislocalization of the hand). (C) Comparison of the human and robot proprioceptive drift normalized by distance (taken from [12]).

#### (ii) The mirror self-recognition experiment

The MSR was originally described as a test for self-recognition in animals [70]. Despite some criticism, it is an interesting framework to evaluate robotic self-recognition capacities [68]. Visual–kinesthetic matching (movement cues) and visual congruency (appearance cues) play a major role. To endow a robot with self-recognition and self–other distinction capabilities, we extended the body localization estimation model with action and visual appearance. The robot learns to predict the observation o given the internal state z, the action a and the noise r: $\hat{o}=m(z,a)+r$, linking the commanded actions with the movement effects observed [13]. The world model m(⋅) function summarizes the learning of the likelihood of the observations, that is, the observation model p(o|z,c), and the dynamics of the state, that is, the state transition model $p(z_{k+1}|z_{k},a_{k},c)$, given its contextual considerations c. Crucially, when learning this world model, the agent can compute the prediction error between the estimated sensory input and the observed one. This error signal is essential to evaluate how likely it is that the robot generated the movement itself. Figure 2 depicts two studies where self–other distinction and mirror self-recognition are evaluated. In figure 2A, the robot learns to differentiate its own generated sensory effects in a mirror through visual–kinaesthetic matching [13]. Learning was modelled with a neural network, which learned the observation model p(o|z,c) and how the action affects the visual input velocities—computed through optical flow. The system infers the state of the robot’s body by optimizing F. If the expectations of the robot match the observed sensory information, then the robot decides that it is itself. In figure 2B, the robot replicates the MSR behaviour, where in the presence of a novel element in the face when facing a mirror, a reaching behaviour emerges [68]. To achieve this, the robot learns its visual representation and how to reach its face using two neural networks. When placing a coloured sticker on the face, the high prediction error (surprise) in that region of the face triggers the reaching mechanism.

**Figure 2.**
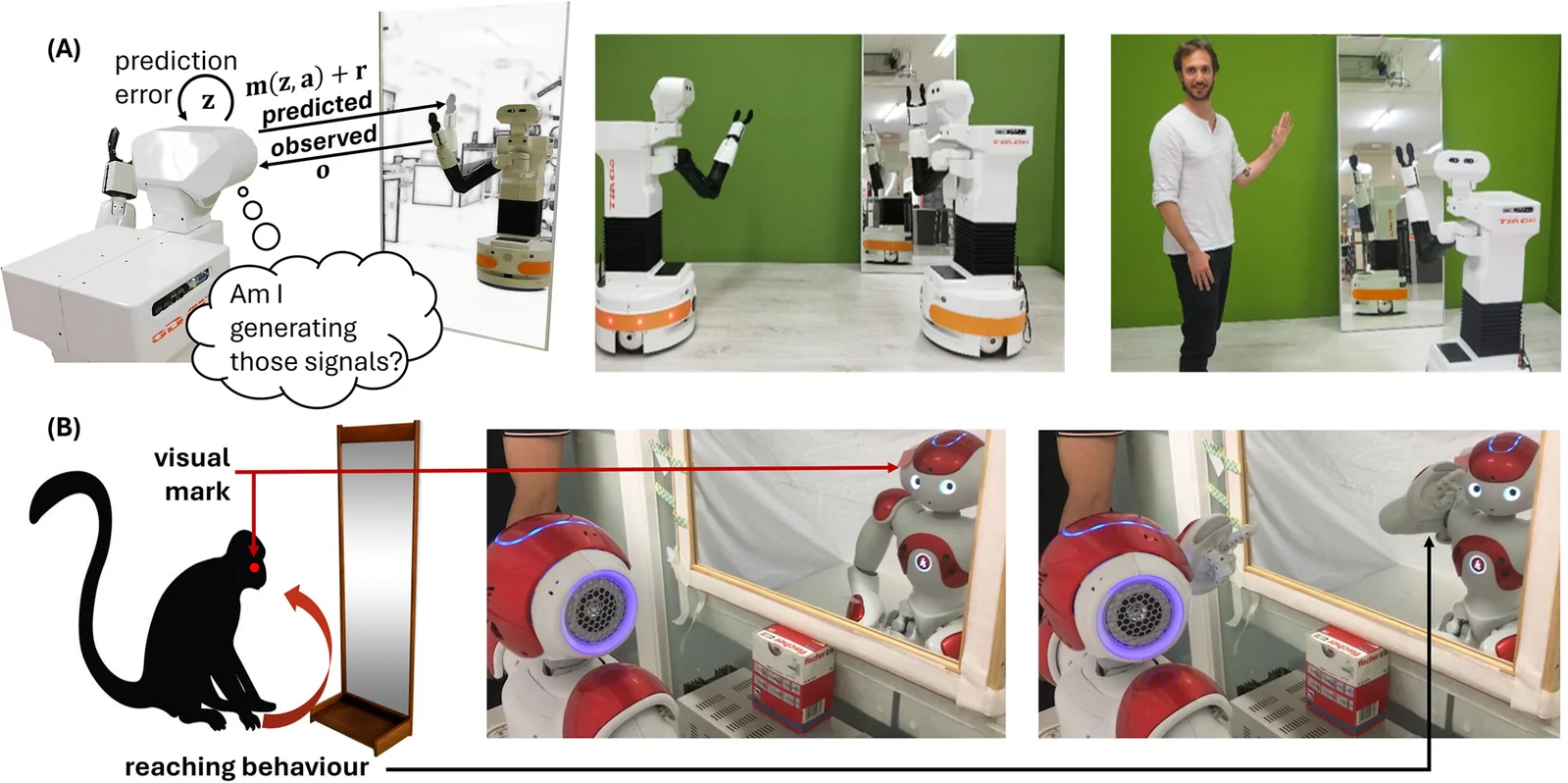
Robotic self-recognition. (A) Self–other distinction through visual–kinesthetic matching by prediction error minimization [13]. The robot learns to predict the sensory input given an action. Thus, when facing the mirror, the prediction error is low (low surprise), and when facing other robots or people, the log-likelihood of being itself drops. (B) Replicating the MSR in a robot [68]. Prediction errors on the visual input, produced by placing a sticker on the robot face, trigger a reaching behaviour that has been learned to touch the face.

### (b) Did I do it?

We already introduced how the robot can evaluate self-generated movement through visual–kinesthetic matching. In principle, this approach can be generalized to effects on the external world. However, agency—the capacity to control our actions and their consequences, together with its subjective experience—goes beyond movement cues and the MSR experiment. Agency may arise when anticipated effects match perceived outcomes during goal-directed actions or uncertainty-reducing actions [76]. Some interesting computational attempts are derived from predictive coding models [77]. Here, causality plays a major role, as does the capacity to anticipate and generalize behaviours. One key finding in studies of agency is that the sense of agency is strongly associated with self-initiated movement. When somebody else moves our arm (passive movement), the sense of agency is greatly reduced or absent [78]. Therefore, the mastering of the sensorimotor contingencies [67,79] and the sense of control [80] is crucial. In essence, the agent needs to understand/learn the consequences of its actions defined by its anticipation capacity. In our model, this is to learn the proper C-POMDP structure, when the contextual focus is on body control. The complexity of building an agentive robot stems from the need to use explicit and accessible abstractions that go beyond the underlying sensorimotor dynamics. Such abstractions enable planning and modulating future actions on different temporal scales. For instance, inductive biases, such as objects [30,81] and event representations [32,33], allow the robot to perform anticipation beyond low-level control signals and implement synthetic self models closer to the theoretical proposals from cognitive psychology [45,82]. Furthermore, it is important to highlight that not only goal-driven behaviours but also uncertainty reducing epistemic action strategies should be incorporated. These are movements or actions that are not captured by the desired goal (or task) directly but have the purpose to reduce state estimation uncertainty [49,76]. While these actions are unconscious, they play a role in anticipation and possibly in agency.

Within this context, the proposed C-POMDP framework offers a useful lens to relate robotic implementations to experimental studies of agency, focusing on observable behaviour under controlled sensorimotor manipulations. However, given an instance of the computational model with the selected inductive biases, how can we evaluate agency in a robot? From the literature, several self-related signatures can be potentially evaluated in a robot or a synthetic agent. One example is the spatial-action effect binding experiment [83,84], where a participant can control a coloured dot on a screen using a stylus pen. Within this effect, objects controlled by the participant produce a mutual attraction of the object’s perceived location and the effective location of the participant’s hand. When presenting an incompatible action–effect condition on the control of the dot on the screen—for instance, by inverting the velocity direction—a decrease in agency is observed. In robots, this corresponds to changes in prediction error [11]. Another example is proprioceptive drift that occurs when we shift the localization of our body parts or the entities that we are controlling. To observe it, we can use the RHI paradigm and also the spatial-action effect binding experiment to employ action as the modulator. In robots, we can induce a proprioceptive drift (mislocalization) of the robotic hand when we manipulate the displacement of the controlled dot. A further example is the temporal binding effect that refers to situations where processing time may be reduced or perceived to be compressed when everything develops as planned, given the desired goal or task [85,86]. In robots, this could only indirectly be compared by assessing processing time and computational complexity.

### (c) Summary and implications

We described how to build a robotic minimal self, with body ownership and agency, through computational modelling. Such robotic implementations provide an ecologically grounded platform to explore and compare theoretical accounts of the self across biological and artificial agents. While there are studies focusing on agency, body ownership and self-recognition in robots, there are still many open questions to be answered, especially concerning the underlying mechanisms. Rather than addressing subjective experience directly, these approaches rely on observable behavioural and sensorimotor readouts, such as proprioceptive drifts under multi-sensory conflict, performance changes during unexpected perturbations and adaptive responses to altered task conditions. Together, these measures offer a pragmatic way to relate computational models, robotic behaviour and experimental findings in the study of minimal self-related processes.

## 4. Open challenges for the robotic self

### (a) Probing the robot’s minimal self

In the previous section, different mechanisms to endow robots with a self have been discussed using experimental implementations of key aspects of the minimal self, such as body ownership and agency. The resulting embodied agents should exhibit self-related properties that can be probed—either by using internal computational measures related to predictive processes as discussed in the previous sections, by information theoretic measures [87,88] or by evaluating the agent behaviour in particular contexts, thereby addressing the challenge of ‘computational (neuro)phenomenology’ [89]. Besides, biologically plausible computational models of the self should also consider their implementation in more realistic neural models of perception and action, such as spiking neural networks [90,91], in order to support the assessment not only of function and computation but also of their inner algorithmic substrate.

#### (i) Embodied Turing test

A behavioural test for a minimal robot self could be based on the famous Turing test for intelligence. In the original version of this test proposed by Alan Turing in the 1950s, a human interrogator had to distinguish a machine from a human based on a text-based dialogue on a computer terminal [92]. One of the major drawbacks of this intelligence test was its intrinsic subjectivity; therefore, it did not measure true AI, but attribution of intelligence, or rather human-likeness. This also holds for sensorimotor Turing test variations that are better suited for measuring agency [93]. Attribution of agency (and the self) to artificial agents can be caused by various factors, including anthropomorphism, appearance and movement [94,95]. Linking the computational measures of an agentive self with behavioural measures could be a way towards probing a synthetic minimal self as well as enhancing our understanding of measures of agency in humans.

Indirectly, we can also replicate human and animal experiments with increasing complexity. Some examples have been introduced here such as the RHI, the MSR and the spatial-action binding effect. However, we can also find many relevant psychophysics experiments for perception and action, such as the anticipatory cross-modal congruency effect [96] for evaluating how anticipation shapes embodied decision-making or the three-dot task for agentic control over external objects [97], and developmental studies for emergent exploratory behaviour [98]. Besides, guiding the requirements of a minimal self [11], a model deployed in a robot that is able to generalize for all experiments would provide a stronger human-like minimal self-related model. Moreover, modelling and replicating these experiments may help to understand disorders of the self (e.g. schizophrenia) [10,99].

#### (ii) Problem-solving

Having a self is useful, especially for interacting with the world. One of the most interesting directions in synthetic self models is that they are actually modelling cognition and connect physical intelligence with complex problem solving. Based on problem-solving in apes [100], we can design a self-disambiguation experiment. An embodied agent is presented with three arms in a visual screen perpendicular to their body axis—two of the arms are fake. Then, a hammer appears over one of the arms. As their proprioception is very noisy, the agent should resolve the problem by raising one of the fingers. This movement identifies the ‘I’ (this is my body). If the hammer is over the owned arm, then the agent removes it. Otherwise, it stays still. The agent then performs an action to disambiguate the scene (reduce uncertainty) and resolve the problem by means of the minimal self: agency (did I do it?) and body ownership (is this my body?). This experiment can be extended to any complex world interaction, especially in human–robot interaction, where a robust self-representation turns into safety, interpretability and accountability.

### (b) Subjective experience and memory of experiences

One of the key challenges of building a robotic self, which includes a deep philosophical and ethical debate, is addressing subjective experience. Human phenomenology cannot be directly addressed in a synthetic self with the current models. Despite this, the computational components constructed in the robot form the basis of the double comparator model [13,101], which is a generalization of the original comparator model [55]. In brief, the first ‘comparator’—low-level (sensorimotor) prediction error mechanisms—allows for handling the body and environmental perturbations and is behind the perceptual and active drifts effects [49,76]. The second ‘comparator’ works as a monitoring system that takes into account prediction congruency (also timing) and self-evidence accumulation to induce subjective body ownership and agency [50].

In RHI experiments, body ownership ratings (through questionnaires) have been captured by the Bayesian causal model [61], particularly when using the synchronicity of the visuo-tactile signals [102]. This means that evidence on the causality of the signals is at least correlated with the subjective experience of body ownership. One of the most interesting proposed mechanisms behind subjective body ownership is uncertainty monitoring [103]—which, in predictive processing accounts, is implemented through estimating the precision parameters. Thus, the second ‘comparator’ would not only be related to congruency matching but also to the uncertainty of the hypotheses handled [104]. Under this framework, in the classical RHI, where there are two hypotheses (I own or I do not own the rubber hand), ownership would be directly modulated by the least uncertain hypothesis given the observations.

In any case, a synthetic self might not have the same type of experiences as a human. An important challenge is to provide the robot with an episodic memory for storing experiences on a temporal self [27,40], therefore allowing for the construction of a ‘narrative’ self [105] and giving the robot an identity beyond the minimal self.

### (c) Social intelligence: understanding agency and understanding others

Humans are social animals. For understanding the mechanisms behind the development of the human (minimal) self, social aspects play a key role. During the first weeks of their lives, infants start to show the ability to distinguish self from others as can be seen in neonatal rooting reflexes [106]. After seven to 12 months of age, they already attribute intentionality to other observed agents, assuming effort-minimizing behaviour and inferring preferences of observed others [107–109]. However, the development of an actual theory of mind (ToM)—the ability to understand that others have intentions and beliefs that can differ from our own—can be shown reliably only at the age of about 4 years [110]. This is probably the case because alternative contextual perspectives that differ from the own perspective and current knowledge need to be maintainable [27]. There are several precursors for a fully developed ToM in human infants, and also suggestions for a ‘social sense’ that could be interpreted as an implicit ToM [111].

The ability to predict the behaviour of others relies on abstract conceptual prediction interactions within social contextual frames, where the frames encode prior assumptions about the knowledge and intentionality of the interacting partners. Accordingly, ToM implies that we can predict and anticipate not only the consequences of our own actions, but also those of others. During development, agents learn to adapt and explore their own body schema, as has been discussed with the example of the RHI and the MSR. First implementations of ToM in artificial agents have implemented consequence engines in multi-agent systems [112], where those systems may be viewed as the social contextual frames without yet making the frames explicit.

These results indicate that the self cannot develop in isolation. With minimal self-abilities in place, it seems to us that the next big challenge lies in the realization of the social self-component, that is, the ability to contrast the perspectives of others with ones own. We propose that this ability requires the activation of alternative (e.g. social knowledge) contextual frames. Given these alternatives, inference chains within the alternative contextual frames will then enable the prediction of the behaviour of others. Interestingly, contrasting behaviour in this way goes hand in hand with the development of more elaborate counterfactual reasoning abilities [27,113,114], which signal deeper human intelligence. Minimal self-related signatures thus seem to lay the foundations for the development of more capable agentive and robotic selves, which will be able to interact with their world and us humans in a much more socially competent and considerate manner.

## Data Availability

This article has no additional data.

## Footnotes

^1^
Ideomotor development is the process where an agent acquires bidirectional action-effect associations [22].

^2^
The world can also transit without the agent actions owing to external causes.

^3^
DFG Priority Programme ‘The Active Self’, http://activeself.de/.

^4^
This is a standard methodology in variational inference in machine learning.

## Theme

One contribution of 17 to a discussion meeting issue ‘From being to identity: analysis and synthesis of the self’.
